# Innovative methodology for flexible monitoring of various bioprocesses by using pre‐existing Raman data coupled with automated transfer learning technique

**DOI:** 10.1002/btpr.70111

**Published:** 2026-02-10

**Authors:** Adèle Schini, Hadi El Radi, Johan Cailletaud, Célia Sanchez, Nathan Gay, Bruno Ebel, Emmanuel Guedon, Laurent Jourdainne

**Affiliations:** ^1^ Millipore S.A.S An Affiliate of Merck KGaA Darmstadt Germany; ^2^ Université de Lorraine CNRS, LRGP Nancy France

**Keywords:** chemometric modeling, instrument calibration, process analytical technology (PAT), Raman spectroscopy, transfer learning, upstream CHO cell culture

## Abstract

This study introduces an innovative approach for the flexible monitoring of bioprocesses using Raman spectroscopy coupled with automated transfer learning. Traditional Raman spectroscopy requires extensive process‐specific calibration, limiting its transferability across different conditions. To address this, we developed an automated method that utilizes the dynamic orthogonal projection (DOP) algorithm to use pre‐existing chemometric models built from one process (“Input Process”) to monitor a distinct process (“Target Process”) which lacked its own Raman models. These new processes conditions varied in terms of culture mode, cell line, media, analyzer, and acquisition settings. This method used spectral data from Target Process to modify the existing spectral data from Input Process, aligning them with the new conditions. The approach was validated on Chinese Hamster Ovary cell cultures, targeting critical metabolic parameters such as glucose, lactate, glutamine, and viable cell density. The results showed that with only one batch used for the transfer, the average relative errors compared to offline values were around 10% for glucose and lactate but remained high for VCD and glutamine. After a second batch to perform the transfer on two batches, the relative errors were further reduced below 10% for most parameters. By effectively transferring models across different processes, this approach minimizes the need for extensive recalibrations, enhancing the efficiency and applicability of Raman spectroscopy in diverse bioprocess environments.

## Graphical abstract

The application of the transfer learning algorithm successfully allowed the use of a model built on process A for real‐time monitoring of process B. This study shows the improvement obtained with this methodology compared to the direct application of the model.

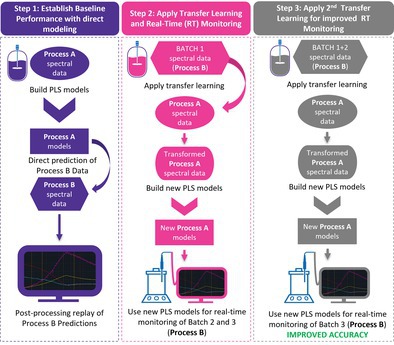

**Abbreviations**
CHO: Chinese Hamster Ovary
CPP: critical process parameters
CQA: critical quality attributes
DO: dissolved oxygen
DOP: dynamic orthogonal projection
DS: design space
EMA: European Medicines Agency
FDA: Food and Drug Administration
IgG: immunoglobulin of type G
LVs: latent variables
mAbs: monoclonal antibodies
PAT: process analytical technology
PC: principal component
PCA: principal component analysis
PLS: partial least square
PP: process parameter
QbD: quality by design
RE: relative error
RMSEC: root mean square error of calibration
RMSECV: root mean square error of cross‐validation
RMSEP: root mean squared error of prediction
Rpm: rotations per minute
RT: real‐time
SNV: standard normal variate
VCD: viable cell density

## 1 INTRODUCTION

Since more than 20 years, the current trend in bioprocessing is going toward the production of recombinant proteins for therapeutic purposes.^1^ Among these molecules of interest, monoclonal antibodies (mAbs) represent nearly half of the market share,^2^ primarily for cancer care^3^, ^4^ and inflammatory diseases.^5^, ^6^ Due to the therapeutic nature of the products, their production processes are subject to rigorous scrutiny. Through the International Conference on Harmonization, regulatory agencies such as the US Food and Drug Administration and the European Medicines Agency have encouraged the adoption of Quality by Design (QbD) principles since the early 2000s, emphasizing the integration of quality and risk considerations throughout the design stages of a process. Thus, the introduction of QbD in the pharmaceutical industry marks a major paradigm shift from a reactive to a proactive model of quality. Rather than focusing solely on quality control of finished products, QbD promotes a holistic approach that integrates quality throughout the product life cycle, from conception to manufacturing and distribution. The main elements of this approach include defining product quality objectives, understanding critical quality attributes (CQA) and the identification of the associated critical process parameters (CPP) thanks to Process Analytical Technologies (PAT), providing a complete understanding of the process.^7^ Among the various PAT tools available on the market, Raman spectroscopy stands out for its non‐invasive and non‐destructive use, enabling real‐time monitoring of biological compounds and rapid detection of variability.^8^, ^9^

Raman spectroscopy is a powerful PAT tool to monitor key metabolites concentrations such as glucose, lactate, and glutamine, as well as protein titer, and viable cell density (VCD).^10^ This monitoring enables to establish the metabolic state of cells in real time and thus opens strategies for feeding control, leading to optimized cell culture processes, and therefore, better product quality and reduced production costs.^11^, ^12^

However, the use of Raman spectroscopy stands on a costly and laborious calibration stage prior to any process integration.^13^, ^14^ Indeed, the intricate highly complex composition of the medium, comprising various types of biomolecules, is reflected in the spectral data where each spectrum represents the sum of unique Raman fingerprints. The main challenge is therefore to build correlation models between spectra and offline reference measurements of sample properties, such as component concentrations. Consequently, building a model requires repeating the same cell culture process several times to gather a consistent amount of data.^15^ Moreover, the spectral data need to undergo preprocessing to remove interference and noise, while carefully selecting relevant spectral variables.^16^ In addition to these demanding model calibration steps, it is crucial to consistently apply chemometric models with the same instrument and under the same experimental conditions as those used for calibration. Variations in cell lines, cell culture media, and broader cell culture conditions lead to distinct spectral data profiles. These discrepancies are reflected by spectral variations that prohibit the direct application of regression models developed under one set of process conditions to another. Such spectral variations hinder the model's ability to generalize across different process conditions, necessitating recalibration or the development of new models for accurate analysis. In this context, users face the challenge of having to restart the model‐building process to incorporate the variability resulting from changes in process conditions. As an example, the team of Santos et al.^17^ identified six factors affecting the Raman signals and categorized them into various groups: “Material, Environment, Process, Measurement, Instrument, and Manpower.” Within these risk categories, eight parameters had a major impact on the Raman signals, most of them falling under the “Material” and “Process” categories.

Pétillot et al.^18^ proposed an approach to lower the impact of instrumental variability between probes and analyzers on model prediction errors. The Kennard Stone Piecewise Direct Standardization (KS‐PDS) method effectively reduced prediction errors, and it was reported that only one batch with a new analyzer integrated into a calibration dataset was sufficient to correct variability and ensure accurate predictions. However, this method only addresses the instrumental variability and not process variability, such as changes in cell lines, media, or measurement conditions. Additionally, methods such as PDS rely on transfer samples, also referred to as standard samples, which must be chemically identical but are measured both on the “master” and the “slave” instrument. In use cases of simultaneous changes of instrument, cell line, media, and feed formulation, all while considering biological variability and uniqueness of each batch, the establishment of standard samples is practically impossible. One possibility being explored is to develop generic models that could be offered directly to users by capturing general trends and relationships in the spectral data, rather than relying on specific instrument characteristics or sample properties.^13^, ^14^ However, challenges still exist in developing truly generic models, particularly in addressing the inherent variability, such as instrumental differences, sample preparation techniques, and environmental factors, potentially impacting the performance.^19^, ^20^ Khodabandehlou et al.^21^ investigated the generic modeling approach utilizing 159 batches with various cell lines and operating conditions to train a convolutional neural network. While the method provided good validation results on a new cell line experiment, it is unclear as to if this new experiment was operated using cell culture media or Raman instruments utilized within the initial training batches. Alternatively, Romann et al.^22^ developed a systematic calibration workflow for perfusion cell cultures, addressing the challenges of low process variability, analyte specificity, and time‐intensive calibration methods. By using a spiking approach with a harvest library from multiple experiments carried out in bioreactor runs, the workflow significantly accelerated model generation while improving chemometric model accuracy for analytes in complex matrices. Finally, Umprecht et al.^23^ propose an unsupervised calibration transfer approach based on preprocessing optimization that minimizes discrepancy between initial and target conditions predicted values, while ensuring acceptable model performance in the source domain. The key advantage is not requiring labeled target spectral data. The method was validated for glucose predictions in a HEK293 cell line culture where the target process differed from the initial process by transgene plasmid, transfection settings, and exposure time. The method was not assessed for any other analyte beyond glucose and not under media formulation or feed formulation and/or feeding strategies changes which might result in conditional or prior shifts by altering fundamental bioprocess chemical compositions and metabolic pathways.

To overcome all the constraints described above, a calibration transfer strategy based on the dynamic orthogonal projection (DOP) algorithm^24^ has been used in this study. The advantages of DOP have been widely described in literature^25^ but its use in bioprocesses remains underexplored. Based on transfer learning, this method could help to optimize the model building for specific key process parameters (PPs), thanks to a reduced number of required cultures, in the case of a new or modified process, while keeping the measurement accuracy expected for a typical model‐building process. The proposed method demonstrates significant proof of novelty by enabling model transfer across a different cell line, a different cell culture media, a different feed solution, a different Raman instrument, and a different acquisition time, all previously mentioned conditions varying simultaneously, with an automated transfer learning that does not require manual hyperparameter optimization. As described by Chen et al.,^26^ transfer learning is a term used in situations where the primary and secondary conditions differ. In such bioprocess cultures where multiple conditions might differ from one process to another, a calibration transfer strategy is well advised to mitigate any resulting model inaccuracy.

Therefore, in the present study, metabolic parameters were targeted for monitoring Chinese Hamster Ovary (CHO) cell cultures with Raman spectroscopy, utilizing an algorithm for the transfer of chemometric models. Models were built on a fed‐batch process, named “Input Process,” prior to this study to monitor glucose, lactate, and glutamine concentrations, as well as VCD. These pre‐existing models were transferred using the DOP algorithm to track a different process (named “Target Process”) which did not have its own chemometric models. Spectral data from one or two cultures of Target Process were used to adapt the initial spectral data from Input Process, modifying them to fit the specific culture conditions of this second process. This approach was tested on batch and fed‐batch cultures of Target Process to cover both applications. An exploratory analysis was carried out to investigate both process data similarities and differences (spectral region, design space), which is required for an effective model transfer. Overall, the approach proposed in this study improved prediction accuracy by over 60% with one transfer learning step and by more than 75% with two transfers, compared to the direct use of pre‐existing models, across all compounds.

## 2 MATERIALS AND METHODS

### 2.1 Input process for Raman models construction

“Input Process” was used to build Raman models for monitoring cultures. Eight fed‐batch processes were run using a CHOZN® cell line (Merck KGaA, Germany), cultivated in EX‐CELL® Advanced medium (Merck KGaA, Germany) within 3‐L bioreactors (Eppendorf, Germany). The medium was supplemented with 0.4% Penicillin/Streptomycin, and feeding was done with a 50%/50% (vol/vol) mix of EX‐CELL® Advanced CHO Feed and Cellvento® 4Feed COMP (Merck KGaA, Germany). Dissolved Oxygen (DO) levels were maintained at 40% air saturation, agitation at 100 rpm, temperature at 37°C, and pH at 7.0 ± 0.1 using CO_2_ and 0.5 N NaOH.

Samples were aseptically collected in triplicate twice a day and analyzed offline using a BioProfile® FLEX2 analyzer (Nova Biomedical, MA, USA) to obtain reference values for VCD, glucose, lactate, and glutamine concentrations.

### 2.2 Target process for transfer of pre‐existing Raman models

The culture medium HyClone™ ActiPro™ (Cytiva, MA, USA) was supplemented with 4 mM L‐Glutamine (Sigma Aldrich, USA) for culturing the CHO DP‐12#1934 clone (ATCC, USA). Cultures were carried out in 2‐liter benchtop bioreactors (Pierre Guérin, France). Six cell cultures were performed: three batch and three fed‐batch as previously described,^10^ fed with CellBoost 5 (Cytiva, USA). DO levels were maintained at 50% air saturation, agitation at 130 rpm, temperature at 37°C, and pH at 7.2 using 0.5 N NaOH and CO_2_.

Twice a day, samples were aseptically collected from the bioreactor and analyzed offline. Samples were centrifuged to separate the supernatant from the cells. Supernatants were analyzed offline for metabolites concentrations, including glucose, lactate, and glutamine using an automatic biochemical analyzer (GALLERY, Thermo Fisher Scientific, USA). Viable cell densities (VCD) were measured using an automatic cell counter (Vi‐CELL, Beckman Coulter, USA).

The Target Process on which the Raman models were transferred differs from the Input Process used by their design, mainly in terms of cell line, culture media, bioreactor setting parameters as well as reference analysis methods (Table 1).

**TABLE 1:** Processes summary table of conditions.

Dataset	Process information					Raman information		
	Samples	Cultures	Culture media	Clone	Feeding media	Integration time (s)	Scans	Time per spectrum (min)
Input process	171	8 fed‐batch	EX‐CELL® Advanced	CHO‐ZN	Cellvento® 4Feed COMP/EX‐CELL® CHO Feed1	30	30	15
Target process (Section 3.1)	16	Batch 1	HyClone™ ActiPro™	CHO‐DP12	NA	20	30	10
	13	Batch 2						
	14	Batch 3						
Target process (Section 3.2)	20	Fed‐batch 1	HyClone™ ActiPro™	CHO‐DP12	CellBoost 5	20	30	10
	20	Fed‐batch 2						
	20	Fed‐batch 3						

### 2.3 Raman acquisitions

The ProCellics™ Raman Analyzer (Merck KGaA) was used with a single probe in single channel mode, with bad pixel and cosmic ray filters activated. The instrument features a 785 nm laser at ~350 mW power at the probe‐tube output. Raw spectral data are acquired over Raman shifts from −80 to 4000 cm^−1^ with 3 cm^−1^ resolution using a back‐thinned CCD; each measurement is the result of an averaging of 30 spectra. The probe was immerged in bioreactors via PG13.connectors, and a lightproof fabric was used to prevent external straylight interference. Laser excitation and data collection were managed by Bio4C® PAT Raman Software (Merck KGaA) via an Ethernet connection between the ProCellics™ Raman Analyzer and the computer. Table 1 summarizes key information on both processes, including the optimal acquisition parameters. Then, the Bio4C® PAT Raman Software was used to associate the reference measurements with their corresponding Raman, creating a consistent dataset for modeling.

### 2.4 Modeling and data analysis

All chemometric analyses were conducted using Bio4C® PAT Chemometric Expert Software (Merck KGaA). The dataset for modeling has been prepared as follows, so that the best quality of data is used for the model building. First, within each 30‐spectra of each single Raman measurement, an automated sorting has been applied using the automatic ejection factor available in the acquisition software. This allowed removing abnormal spectra, if any, eliminating undesired spectra that could bias regression model development. Then, the spectra were averaged to obtain a clean spectrum for each single Raman measurement. Then, those spectra were preprocessed following three steps using (1) Standard Normal Variate (SNV) normalization^27^ calculated on the water region between 3100 and 3700 cm^−1^ to reduce inter‐batch variability and correct for variations in optical scattering. Next, (2) Savitzky–Golay first derivative^28^ (second order polynomial and 5‐point fitting window) has been applied to decrease the fluorescence effect and accentuate small spectral contributions. Finally, (3) spectral selection has been applied to use only the regions between 350–1775 cm^−1^ and 2800–3000 cm^−1^ for the modeling; those regions are selected to capture the organic spectral fingerprints of the main compounds.^13^, ^14^

To gain a comprehensive understanding of the data and effectively model the similarities and differences between both processes, a tailored data analysis approach was carried out. This approach includes Principal Component Analysis (PCA)^29^ to first explore qualitatively the spectral dataset. Then, Partial Least Squares (PLS) regressions^30^ to correlate the Raman spectra with reference concentrations of target analytes using latent variables, and finally, DOP^24^ was used to minimize the effects of various process conditions between multiple datasets.

#### 2.4.1 Principal component analysis (PCA)

PCA is a technique recognized as the basis of chemometrics.^31^ The algorithm was mainly developed to reduce the dimensionality of a multivariate dataset by capturing the underlying structure of the correlations between the different variables. In the context of Raman spectroscopy, variables refer to the Raman shifts that constitute a Raman spectrum and provide valuable information about the chemical composition of the sample.

PCA decomposes the initial data according to new variables called principal components (PCs). The PCs were calculated from a linear combination of the initial variables to progressively explain all the information contained in the original dataset.^29^, ^32^ In addition, the PCs were built in the sense that no information contained in one PC was present in another. A data matrix could thus be decomposed into as many PCs as it has distinct axes of variability while ensuring that each additional calculated PC could explain the residual information not addressed in the previous PCs.

Careful examination of the PCs could lead to a better understanding of the different variation sources. During the modeling step, the PCA algorithm was initially used for data visualization and the identification of clusters or trends, also including outlier detection.

#### 2.4.2 Partial least squares (PLS)

Partial least squares (PLS) regression was used in this study as a quantitative statistical method to relate the Raman spectra and the different metabolites to be monitored. PLS regression operates by finding linear combinations of the original variables, Raman shifts in this case, which are called “latent variables” (LVs). The method was built in a way that each successive LV explains the maximum remaining variance in the Raman spectra while exhibiting the highest possible covariance between the scores of the Raman spectra and those of the metabolites.

The optimal number of LVs for each PLS model was selected based on minimizing the root mean square error of calibration (RMSEC) and the root mean square error of cross‐validation (RMSECV) while maximizing the cumulative explained variance (R^2^Y) and the cumulative predicted variance (Q^2^Y). The built models were used for real‐time monitoring of cell culture processes. The monitoring accuracy was evaluated by calculating a Root Mean Squared Error of Prediction (RMSEP) and a relative error (RE)^33^, respectively given by the Equations (1) and (2).
(1)

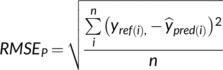

(2) $$\mathrm{RE}=\frac{{\text{RMSE}}_{P}}{y_{\max}}\times 100$$

where ${\hat{y}}_{\text{pred}\left(i\right)}$ is the concentration value calculated by the PLS model for sample i, $y_{\mathrm{ref}\left(i\right)}$ the associated reference value, n the number of samples in the selected dataset and $y_{\max}$ the maximum reference value of the concentration range for the considered parameter.

#### 2.4.3 Dynamic orthogonal projection (DOP)

With the aim of comparing process conditions, the DOP algorithm aligns data from the Input Process with the Target Process by projecting both into a common orthogonal space as described by Fonseca Diaz et al.^34^ This shared space effectively filters out variability that does not pertain to the metabolites of interest, thereby enhancing the robustness of the resultant PLS models against discrepancies between the two processes.

**FIGURE 1:**
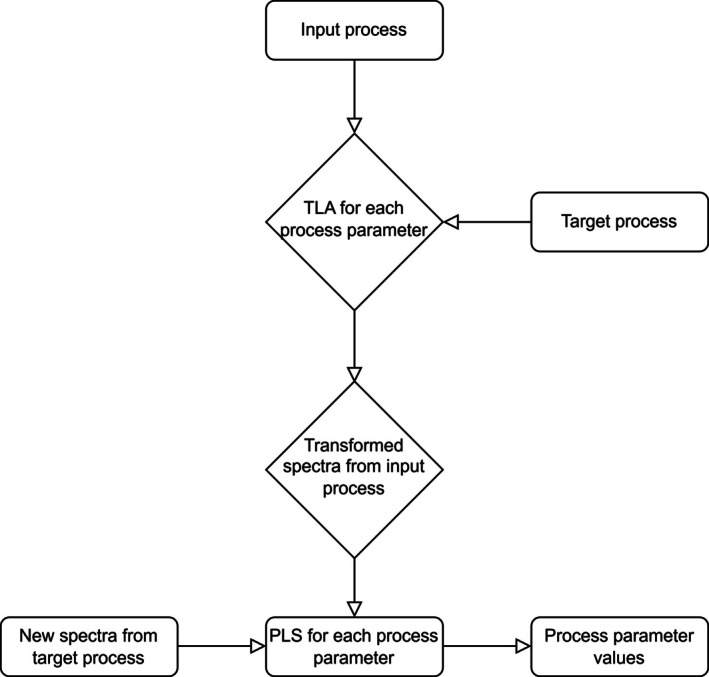
Flowchart representing the data processing for the application of TLA.

The DOP algorithm takes data from both Input Process and Target Process, comprising preprocessed spectral and metabolite information. The method was utilized within the commercially available Bio4C® PAT Chemometric Expert software (Merck KGaA) which utilizes DOP within a generalized Transfer Learning Algorithm (TLA) software module. As illustrated in Figure 1, the algorithm computes a series of DOP projections according to each metabolite to quantify, which are then incorporated into multiple PLS regression models.^24^ The TLA software module automatically optimizes DOP critical hyperparameters described by Zeaiter et al. which are customized to each metabolite.

## 3 RESULTS AND DISCUSSION

### 3.1 Transfer learning of Raman data acquired from fed‐batch (input process) into new process Conditions 1 (batch target process)

Initially, the study was focused on the transfer of pre‐existing regression‐built models based on a fed‐batch type Input Process to a batch‐type Target Process. In mAbs production processes, batch mode is the first and simplest stage before fed‐batch, that is, the addition of nutrients to promote cell growth and mAbs production. In this way, the robustness of transferred Raman models was assessed while minimizing the volume of media used.

#### 3.1.1 Exploratory analysis

The transfer of pre‐existing models from one process to another holds significant promises for a faster implementation of Raman spectroscopy for real‐time monitoring. However, ensuring the efficacy of such model transfers requires exploratory studies of the spectral data. These studies were performed to assess the compatibility and similarities between datasets. They aimed to validate the feasibility of transferring models, despite potential variations in the experimental conditions.

##### Process parameters study

The study of process parameters (PPs) is crucial as the automated transfer learning technique depends on existing data to aid in constructing models for the target process. To achieve optimal transfer learning, the PPs of the target process must fall within the range covered by those of the input process. While the distribution of values within this range influences the quality of transfer learning, it does not restrict its applicability. The primary limitation is that the method cannot extrapolate beyond the historical or input data ranges.

**FIGURE 2:**
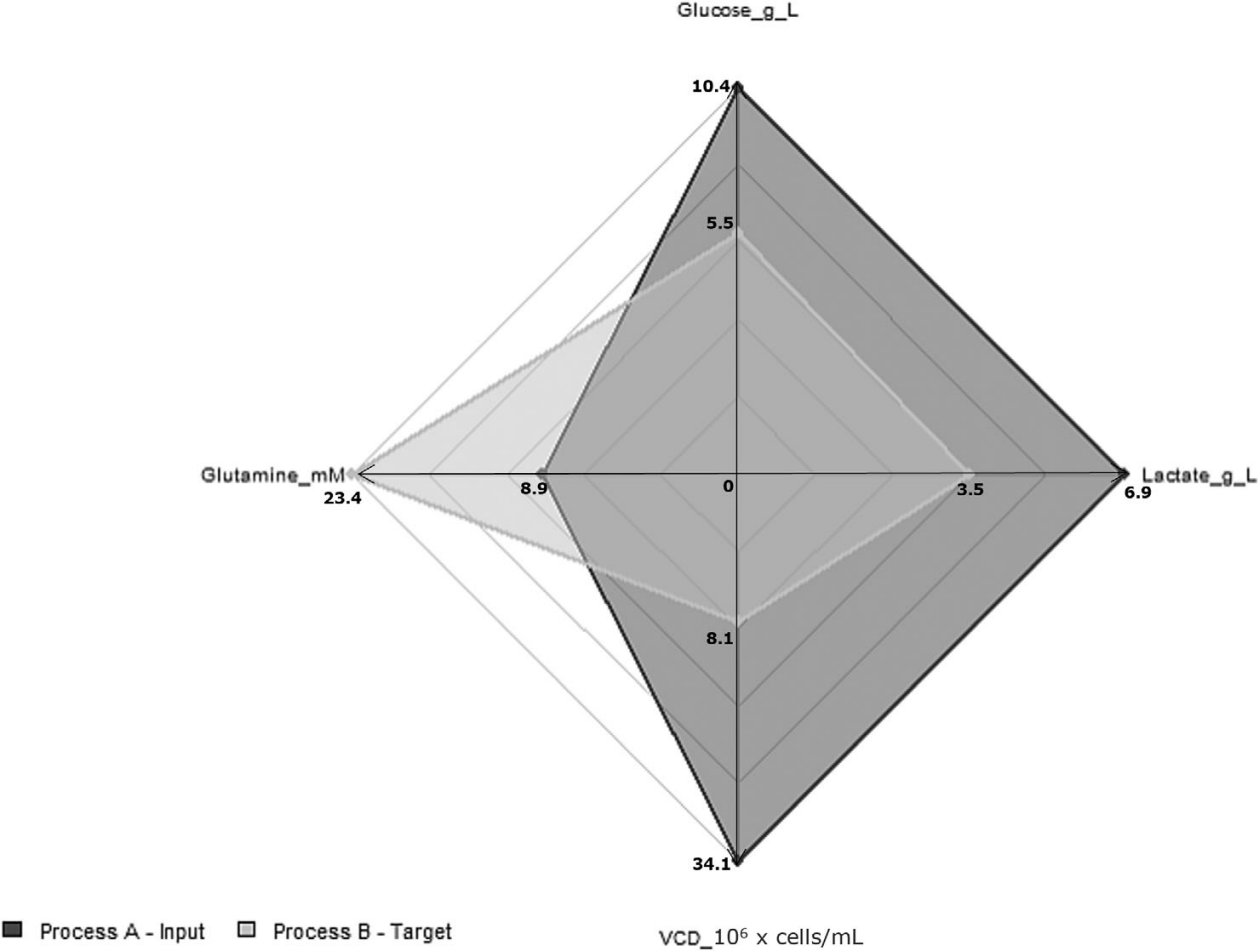
Radar chart representing the ranges of measured concentrations for input process and target process for the batch study.

In Figure 2, values displayed on each axis correspond to the areas covered by metabolite concentrations for each process. Input Process surfaces of glucose, lactate, and VCD concentrations overlap Target Process ones, indicating a good feasibility of the model transfer. However, it is not the case for glutamine concentrations, with Target Process exhibiting higher levels in the culture medium prior to bioreactor inoculation. This result provided an initial indication of the feasibility of model transfer to monitor glucose, lactate, and VCD but not for monitoring glutamine. The analysis was further detailed into the Raman spectra to ensure that the spectral signatures were sufficiently similar to consider model transfer.

##### Spectral analysis

**FIGURE 3:**
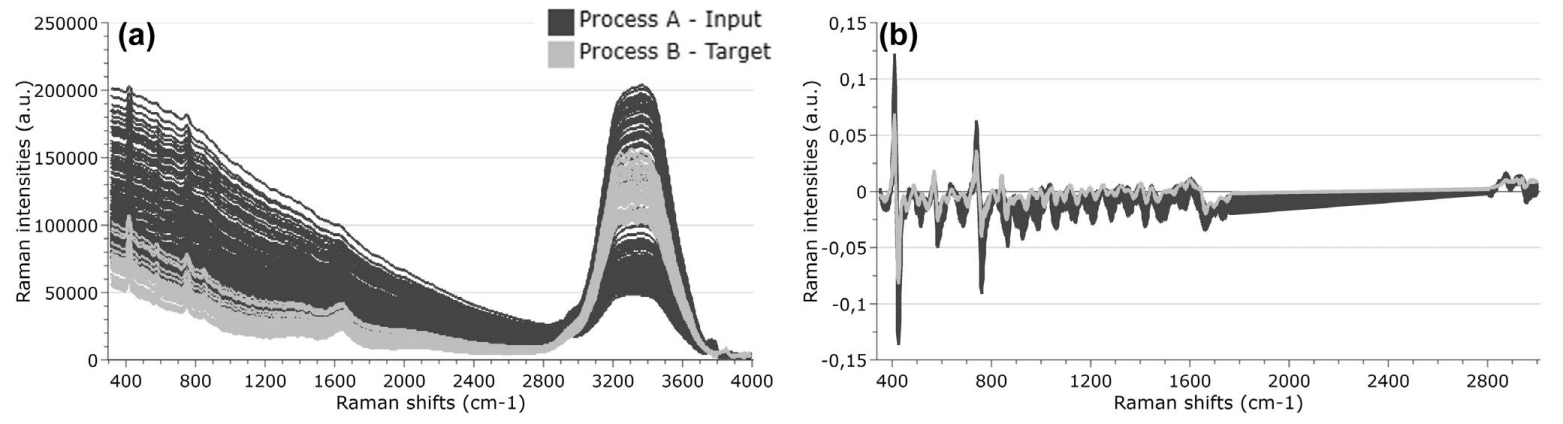
Representation of raw (a) and preprocessed (b) Raman spectra of both processes. Data from Input Process are colored in black and Target Process in gray.

The Raman spectral data acquired for both processes are displayed in Figure 3. The raw signal intensities for Target Process (Figure 3a) were generally lower than those for Input Process (Figure 3a), primarily because of a reduced acquisition time.

The preprocessing described in Section 2.4 and shown on the data in Figure 3b was an essential step to remove a first layer of variability between the two processes. Upon initial interpretation of the spectral data, it was observed that despite variations in sensor instrumentation, cell culture media, and cell lines, both processes exhibited similar Raman spectral signatures. Indeed, the various metabolites of interest are common factors between both processes, albeit present in varying concentration ratios. To understand the origin of the variations still visible in the Raman intensities, PCA was performed on the two datasets.

##### Principal component analysis (PCA)

As described in Section 2.4.1, PCA facilitates data interpretation especially in the case of spectroscopic data, where there is often a high degree of collinearity among different Raman shifts. The score plot simplifies visualization and allows distinguishing differences or similarities within various samples of the dataset. Such observations can be contextualized within the experiments to provide a more comprehensive view of the data.^35^

**FIGURE 4:**
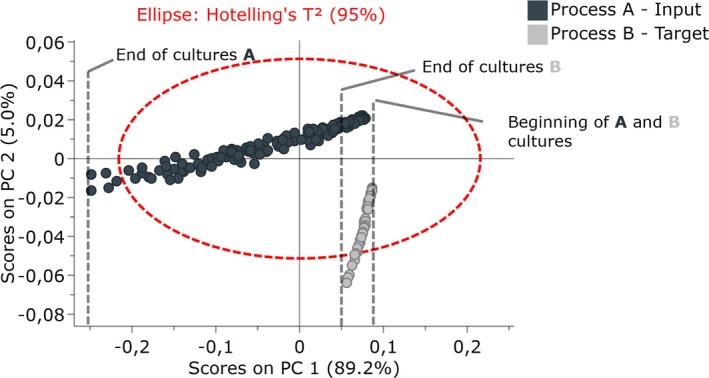
PCA score plot illustrating the variation between Input Process (black points) and Target Process (gray points).

The PCA score plot (Figure 4) illustrates that the spectral variability of samples from the input process and the target process in batch conditions are explained with the two first principal components (PC1 and PC2) accounting for 94% of the total variance in the dataset. The samples from Input Process and Target Process do not overlap in this score plot, but almost all samples remained within the Hotelling's T^2^ ellipse,^36^ indicating that there were significant distinct variations captured by these components while still maintaining a 95% confidence interval. The proportion of variance explained by PC1 (89.2%) suggested that this component was the primary driver of variations in the dataset. As visually illustrated in Figure 4, PC1 variability is highly driven by the elapsed time of cultures A. The first spectral acquisition collected on each culture conditions displayed a very similar score value on PC1 (0.08 for Input Process and 0.09 for Target Process) but the evolution across PC1 is highly different. After careful look at both datasets, the primary drivers of PC1 were both cell density growth and antibody yield (data not shown). While PC1 did not spatially discriminate the two processes, it could be observed that Input Process had much more variations in this axis compared to Target Process. This could be attributed to the fact that Input Process was a fed‐batch of approximately 2 weeks duration with high cell density growth (up to 34.1 × 10^6^ cells/mL) while Target Process was a one‐week batch culture with low cell density (up to 8.1 × 10^6^ cells/mL). Target culture in batch mode had a very poor productivity and growth in comparison, which explains its limited evolution on the PC1 axis. However, PC2 (5%) highlights a significantly different score value for the first data point of each process (0.02 for Input Process and −0.01 for Target Process), and this difference extends over process time. This observation indicated that the discrepancies between the two process conditions were more visible through PC2, which is representing 5% of variability and with glutamine variations of target process as a significant contributing process variable for PC2 (data not shown).

The findings from this PCA analysis were consistent with expectations, as distinct processes typically generate different Raman signatures.^17^

This preliminary study comprising process parameter analysis, spectral analysis, and PCA analysis provided a better characterization revealing more insights into compatibilities and divergences of both datasets. Thus, it was demonstrated that it was possible to confidently consider transferring existing Raman models created with Input Process to a different Target Process to monitor parameters that share the same design space (DS).

#### 3.1.2 Quantitative analysis

Changing process conditions such as, in this study, the media and the cell line would require acquiring enough data of batch cultures in the new process conditions to train new PLS models. Before reaching the desired goal, which is real‐time process monitoring, a lot of resources were dedicated to build models. Quantitative analyses demonstrated the use of transfer learning technique to have a model ready for monitoring the new Target Process conditions, in a fast manner, thus reducing the efforts that were needed to obtain accurate and reliable process quantifications.

#### 3.1.3 Direct monitoring versus transfer learning

**FIGURE 5:**
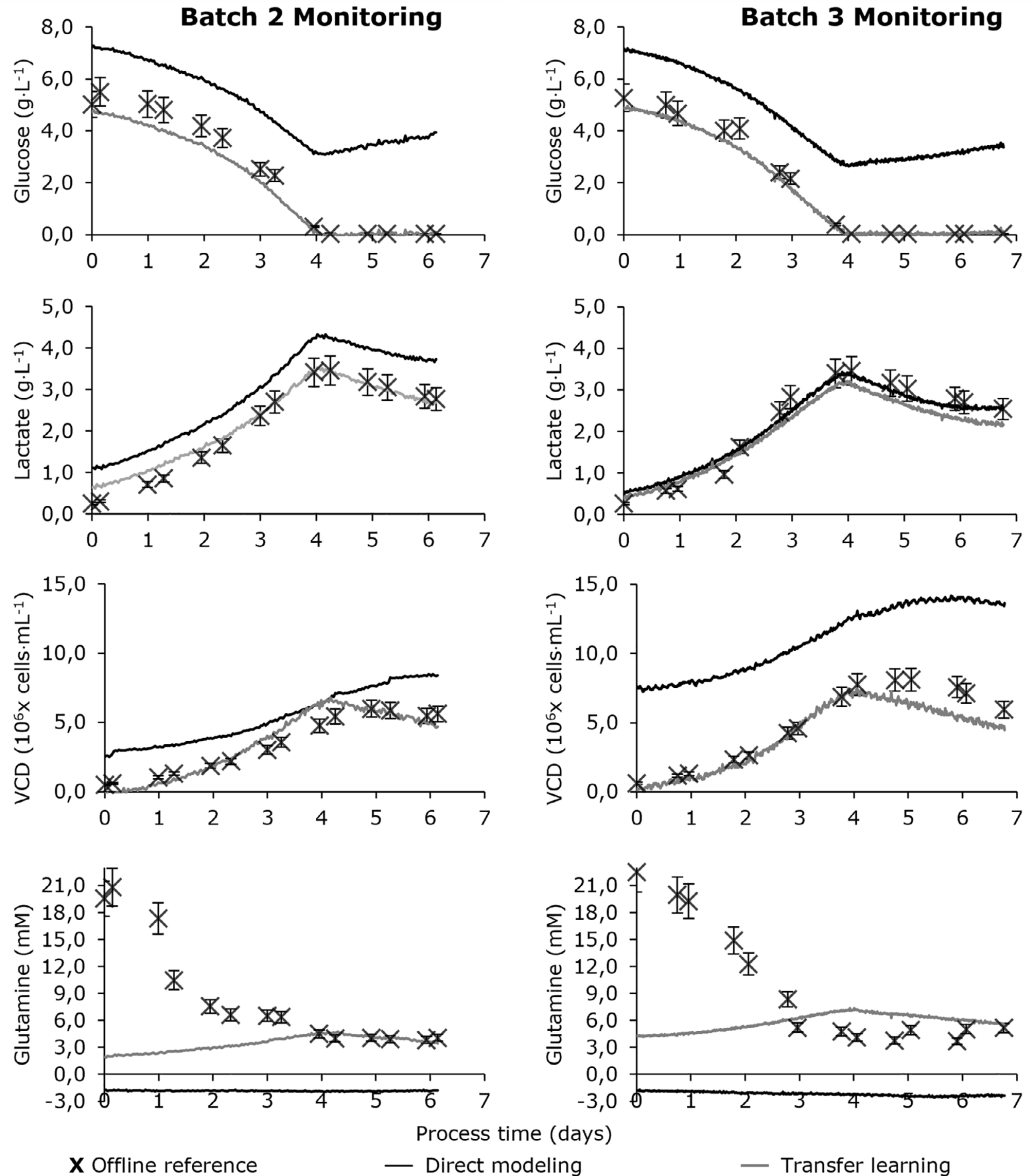
Monitoring Batch 2 and 3 (Target Process) using direct modeling (black curve) and the transfer learning method (gray curve). Crosses represent offline values. Error bars are based on the tolerance of the analyzer, that is, ± 10%.

In a first step, the PLS Raman models created from the Input Process were directly applied in monitoring of the Target Process (Batch 2 and 3) without applying any transfer algorithm. This first step enabled a baseline performance level to be defined, so that the predictive accuracy of the models after transfer learning could then be assessed (Figure 5, “Direct modeling” curve). The data obtained from direct modeling using the Input Process models were compared to the offline reference values obtained for each experimental collected sample (Figure 5, crosses). Overall, the prediction trends for glucose and lactate appeared similar to offline data, with average offsets of 2.5 and 0.8 g L^−1^, respectively, demonstrating that direct modeling could offer promising accuracy. Considering a potential strategy to eliminate this offset could further enhance the model's precision. However, this was not the case for VCD or glutamine, where the predictions do not align with the offline measurements. Table 2, summarizing the various statistical errors obtained for the direct modeling, indicates a mean RE of ~50% for glucose monitoring, 15% for lactate, 60% for VCD, and 46% for glutamine, considering Batches 2 and 3 combined. Considering that the offline analyzer had a measurement tolerance of ~10%,^37^, ^38^ the direct use of the Input Process data to monitor the Target Process did not show sufficient prediction accuracy. It is important to mention that the model from Input Process based on eight fed‐batches is routinely used to monitor new fed‐batches in the same process conditions with excellent accuracy (data not shown). Rather, the emphasis of the direct monitoring results is to highlight the monitoring limitations when trying to use this otherwise working model on a new set of process conditions. Consequently, the batch 1 spectral data from the Target Process were used to transform those from Input Process and subsequently create new adapted PLS models.

**TABLE 2:** Summary table of performances of different modeling strategies (direct or transfer learning) as applied to Batches 2 and 3.

Parameter	Monitoring batch	Range	Direct modeling		Batch 1 used for transfer learning		Batch 1 + 2 used for transfer learning	
			RMSEP	RE	RMSEP	RE	RMSEP	RE
Glucose (g L^−1^)	Batch 2	0–5.51	2.69	49%	0.52	9%		
	Batch 3	0–5.28	2.64	50%	0.31	6%	0.12	2%
Lactate (g L^−1^)	Batch 2	0.25–3.46	0.80	23%	0.21	6%		
	Batch 3	0.26–3.46	0.21	6%	0.33	10%	0.16	5%
VCD (10^6^ × cells mL^−1^)	Batch 2	0.48–5.99	2.10	35%	0.70	12%		
	Batch 3	0.63–8.12	7.00	86%	1.10	14%	0.41	5%
Glutamine (mM)	Batch 2	3.83–20.83	11.92	57%	8.46	41%		
	Batch 3	3.69–22.54	8.06	36%	8.37	37%	6.42	29%
	**>10% RE**	**<10% RE**						

The efficacy of transfer learning was validated across two new subsequent batches to ensure repeatability and assess the robustness of this approach. After the application of transfer learning on the preprocessed spectra, the newly created models were used for real‐time monitoring of batch 2 and 3 (“Transfer learning” curves, Figure 5). RMSEP and RE values were calculated to estimate transfer learning performance compared to direct modeling (Table 2). All RMSEP values were significantly reduced except for the monitoring of glutamine in Batch 3. The RE remained high for the monitoring of glutamine, indicating a poor performance in the transferability of models for this compound. The fact that glutamine was outside the DS (Figure 2) in relation to Input Process explains the difficulty in the method which was not capable of extrapolating beyond the input process range of values. This demonstrated that transfer learning effectively was able to adapt Raman models to new process conditions, outperforming direct modeling with pre‐existing models in accuracy and reliability. While significant improvements were observed for glucose and lactate monitoring, further enhancements are needed for VCD and glutamine monitoring, justifying the need for a new transfer learning application in order to improve the models for these compounds.

#### 3.1.4 One‐batch versus two‐batches transfer learnings

**FIGURE 6:**
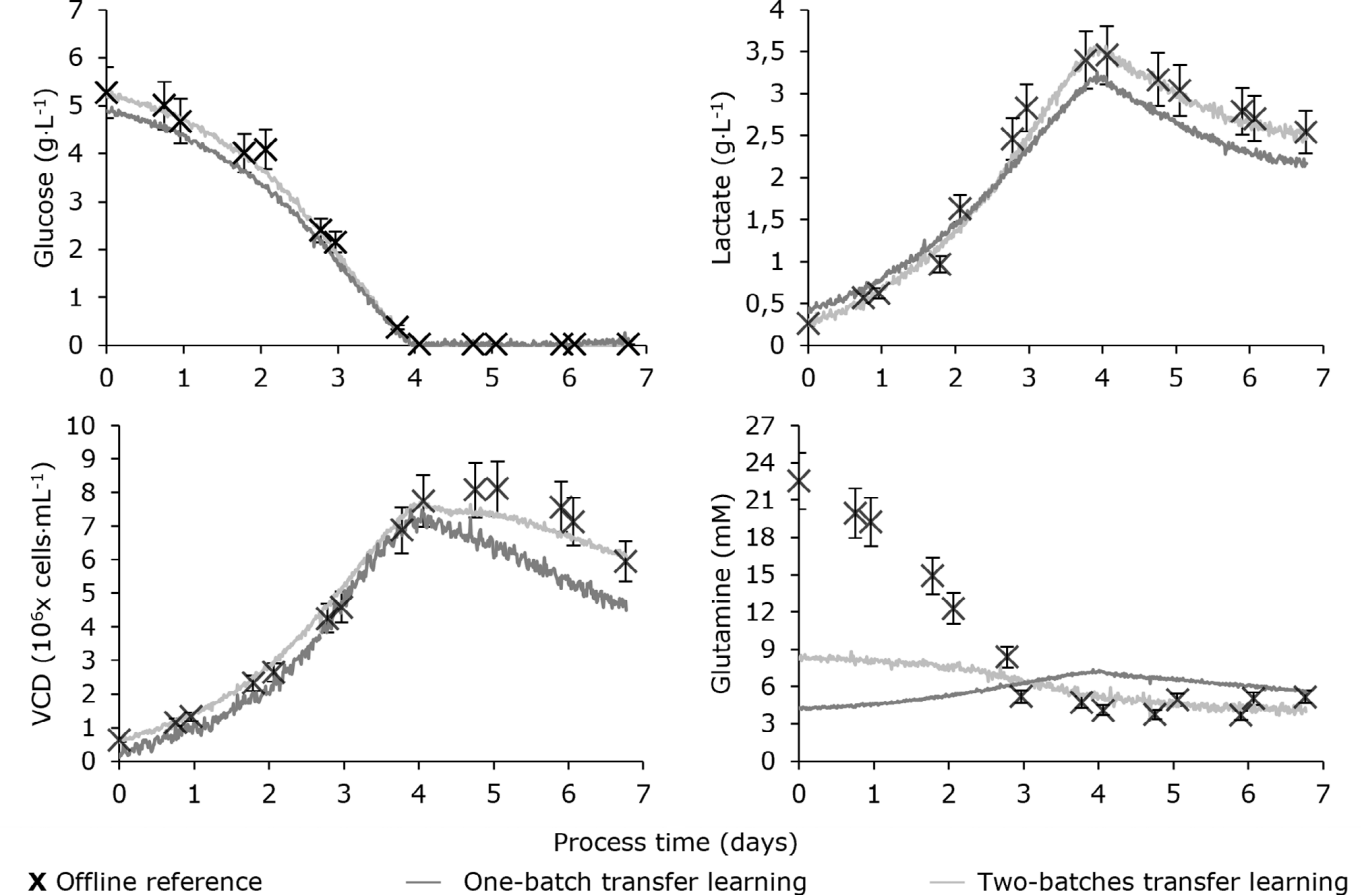
Monitoring of Batch 3 with models (from Input Process) modified by transfer learning using Batch 1 (dark‐gray curve) and using Batches 1 and 2 (light‐gray curve). Crosses represent offline values. Error bars are based on the tolerance of the offline analyzer, that is, ± 10%.

To improve the accuracy of VCD and glutamine monitoring from the Target Process, a new transfer learning was carried out. This new transfer learning is also based on the same input process data but it utilizes data from Batches 1 and 2 combined instead of just 1 to modify the spectral data from the Input Process. The newly created models were then used for real‐time monitoring of Batch 3. The results obtained at the end of the culture were then compared to those obtained in parallel with the models previously transferred using only Batch 1 (Figure 6, Table 2).

Overall, this transfer based on the first two batches improved the accuracy of the models for each of the four monitored parameters. The RE were reduced by almost threefolds for all compounds compared to the single batch transfer learning method. Only the RE for glutamine remained above 10%, which is the desired upper limit. But looking closely into the glutamine monitoring dynamics as outlined in Figure 6, the Raman monitoring line is well aligned with offline glutamine values below ~9 mM after day 3, and 8.9 mM corresponds to the upper value of the glutamine range in the input process. The method still showed improved accuracy, but within its limitations to extrapolate resulting in an overall high error across the whole process duration. The various RMSEP values were also reduced by ~50% for all compounds, except for glutamine. This confirms that the concentration ranges for the chosen compound must be within the same DS, as a prerequisite for effective transferability. To improve glutamine monitoring, it could be considered to create a specific PLS model using only the data from Batches 1 and 2 from the Target Process, which would then consider a calibration range beyond glutamine levels of the input process. Beyond the DS challenges, the inaccuracy of glutamine monitoring by Raman spectroscopy has already been reported in other studies,^8^, ^39^ mainly due to its molecular structure being close to that of other amino acids and resulting in overlapping spectral signals.

When using Raman spectroscopy for glucose and lactate monitoring, a single transfer may be sufficient if a very high measurement accuracy is not required, specifically below 5% RE. Regarding VCD, the single transfer worked less effectively, particularly at the end of the culture. A possible hypothesis to explain this observation is that a decrease in cell viability (see Figures S1 and S2) led to cell lysis and, therefore, a release of internal cellular compounds in the medium and resulting in an increase in fluorescence and noise. A confidence index for the measurement could be provided to the user based on the growth phase, or alternatively, an uncertainty measure could be included alongside the displayed values to assess measurement reliability.^40^ Furthermore, monitoring VCD with Raman spectroscopy relies on an indirect measurement of the cells' metabolic activity, leading to variations in proteins, nucleic acids, lipids, and carbohydrates in the process. Thus, unlike dielectric spectroscopy, this approach does not directly link the measurement to the live cell state. This undirected quantification of VCD makes it a more challenging parameter to model compared to glucose, for example.

The dual‐transfer approach using data from two batches resulted in better predictions compared to the single‐transfer method and could be utilized for process control based on Raman monitoring. Nevertheless, the above results were obtained on a batch process. Modification of the process from batch to fed‐batch cultures induces spectral variations because of the several feed additions across process duration that were not previously incorporated into the transfer learning process, thus degrading the accuracy of models developed under batch conditions (data not shown).

### 3.2 Transfer learning of Raman data acquired from fed‐batch (input process) into new process Conditions 2 (fed‐batch target process)

Similarly to what was established for batch culture transferability, three fed‐batch cultures were carried out. The first fed‐batch was used as an input of the DOP algorithm to transfer the Input Process data, and the following two fed‐batches were run to evaluate the effect of transfer learning. The study of the DS for the fed‐batch cultures showed similar outputs to the previous batch section, including higher glutamine values in the target process fed‐batch runs compared to the Input Process (data not shown as it is redundant with Section 3.1.1 and does not provide further insights into the proposed methodology).

#### 3.2.1 Direct monitoring

**FIGURE 7:**
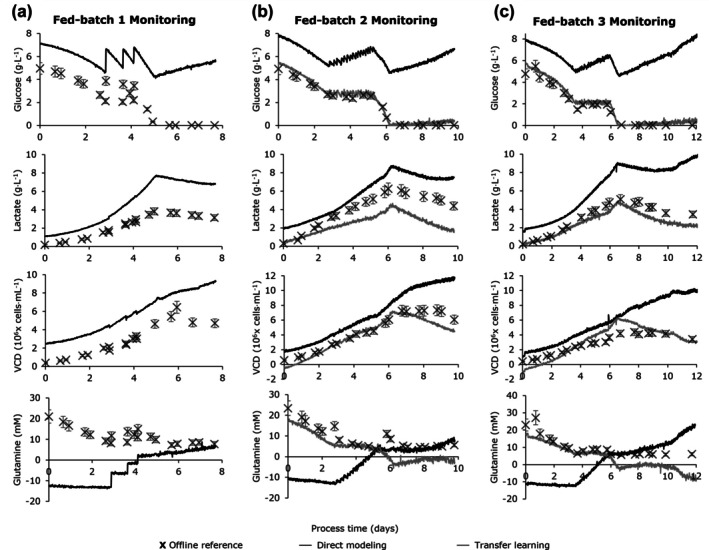
Monitoring of 3 fed‐batch cultures using PLS‐models built from Input Process fed‐batches with direct modeling (black curve) or transfer learning methods (gray curve). (a) Direct modeling of Fed‐Batch 1. (b) Direct modeling and modeling after one transfer learning. (c) Direct modeling and modeling after two transfer learnings. Crosses represent offline values. Error bars are based on the tolerance of the analyzer, that is, ± 10%.

The direct application of pre‐established models generally did not provide accurate monitoring of the fed‐batch process. Although the overall trend of the curves aligned with the variations in offline values, the models showed an average RE of 67% across different parameters (Figure 7a). The significant addition of glucose following the feeds was clearly detected by the Raman models throughout the various kinetics, but the values still exhibit a 70% RE compared to the reference offline values. However, the estimated values for glutamine were negative for nearly 4 days, indicating a substantial discrepancy in the models for this compound, likely due to incompatible DS, as mentioned above in the Exploratory analysis section. The high RE values reflected the poor performance of these models, otherwise accurate when used in similar process conditions as those of the input process. The results underscore a need for the transfer learning method to obtain satisfying accuracy (Table 3).

**TABLE 3:** Summary table of monitoring performances for direct modeling and transfer learning techniques applied for monitoring three fed‐batch cultures.

Parameter	Monitoring batch	Range	Direct modeling		Transfer learning	
			RMSEP	RE (%)	RMSEP	RE (%)
Glucose (g L^−1^)	Fed‐batch 1	0–4.97	3.5	70%		
	Fed‐batch 2	0–4.92	3.68	75%	0.28	6%
	Fed‐batch 3	0–5.49	3.75	68%	0.34	6%
Lactate (g L^−1^)	Fed‐batch 1	0.17–3.81	2.56	67%		
	Fed‐batch 2	0.29–6.25	1.53	24%	1.45	23%
	Fed‐batch 3	0.20–5.08	2.52	50%	0.50	9%
VCD (10^6^ × cells mL^−1^)	Fed‐batch 1	0.40–6.45	2.64	41%		
	Fed‐batch 2	0.80–7.34	1.83	25%	0.54	7%
	Fed‐batch 3	0.60–7.13	2.19	50%	1.28	31%
Glutamine (mM)	Fed‐batch 1	7.32–21.1	18.4	87%		
	Fed‐batch 2	3.63–23.4	19.0	81%	5.95	25%
	Fed‐batch 3	5.75–27.2	20.1	74%	5.41	20%
	**>10% RE**	**<10% RE**				

#### 3.2.2 Direct monitoring versus transfer learning method

A transfer learning approach was first applied using data from Fed‐Batch 1 (Target Process) applied to the input process data. This resulted in PLS models calibrated on filtered input process (as generally described in the flowchart in Figure 1) which were then used to monitor two new fed‐batches (see Figure 7b,c for fed Batch 2, and Figure S3 for fed Batch 3) demonstrating improved accuracy compared to direct modeling. This improvement is particularly significant for glucose and VCD, where the respective RMSEPs were reduced by 13 and 3 times, and the REs by more than 12 and 3 folds. An improvement was also observed for glutamine monitoring with one transfer, but the RE remained high, above the 10% tolerance threshold. Nevertheless, the enhanced models for lactate did not improve the accuracy compared to the direct modeling approach. This can be explained by the fact that Fed‐Batch 1 used for the transfer had lower offline lactate values compared to Fed‐Batch 2. As a result, the model monitored better the Fed‐Batch 3, which displayed a comparable lactate kinetic production compared to Fed‐Batch 1. The lactate inaccuracy for Fed‐Batch 2 highlights a limitation of the method when the subsequent runs post transfer learning have significant gaps (~35% more lactate production in Fed‐Batch 2 compared to Fed‐Batch 1). To further evaluate the benefits of transfer learning within an appropriate DS, it would be valuable to compare a model transferred from Fed‐Batch 1 to a model transferred from Fed‐Batch 2, applied to Fed‐Batch 3. This comparison could highlight the importance of selecting the right DS for transfer learning. Although not the main objective of this paper, it is worth mentioning that Fed‐Batch 2 and Fed‐Batch 3 were controlled in glucose value during several days by utilizing real‐time glucose monitoring values from the Raman. Every time the Raman monitored value would go below a predefined threshold value, a bolus addition would be triggered to maintain glucose values at the desired level. This transfer learning methodology gives scientists access to efficient and precise (Figure 7) glucose control loops utilizing just one fed‐batch of training.

To attempt a better monitoring of lactate, VCD, and glutamine concentrations, a new transfer was performed using data from Fed‐Batch 1 and 2 combined to modify the input process data and the new PLS models obtained were applied to Fed‐Batch 3 monitoring. This had the beneficial effect of reducing the RE below 10% for lactate monitoring and slightly improving the accuracy of glutamine tracking. However, this second transfer introduced variability in monitoring VCD, increasing the model's imprecision. Indeed, as explained above with the case of lactate, the DS of Fed‐Batch 1 and 2 were similar in terms of VCD values, allowing for a high‐quality transfer between both datasets. However, Fed‐Batch 3 had lower offline VCD values compared to Fed‐Batch 1 and 2, making the model created from these two datasets less accurate. This result highlighted the importance of reliable offline VCD evaluation methods when obtaining values for model construction. In this study, both analyzers relied on trypan blue staining and image analysis software. Consequently, variations in grayscale parameters used during image analysis could result in discrepancies between measurements for the same sample. Measuring the same sample with both reference devices for comparison would have provided valuable insight into potential differences in VCD assessment across analyzers.

The aim of the transfer learning method is to facilitate bioprocess monitoring implementation in realistic conditions when users acquire their first couple of runs. As the first runs are used for transfer learning, they could be representative of a typical process or not, as observed in Fed‐Batch 3; the VCD profile was different than the two previous fed‐batches. These variations are part of the biological variability and the real‐life challenges of the industry.

Finally, the proposed method facilitates implementation when moving into a new set of process conditions such as a new media, feed, cell line, or all simultaneously by drastically reducing the number of runs required before moving into a sufficiently accurate monitoring phase. This methodology does not aim to replace traditional, robust models based on large datasets from these new conditions. Instead, it can be implemented early in the process development phase, accelerate validation phases, and allow users to benefit from in‐line sensing process knowledge for optimization before they accumulate sufficient data for conventional models.

## 4 CONCLUSION

This study has successfully demonstrated the feasibility and effectiveness of transferring pre‐existing chemometric models between similar processes using the DOP algorithm as a transfer learning method when the target monitoring values are included within the DS of the existing model. By applying this method, the accuracy of Raman spectroscopy models for monitoring key metabolic parameters (glucose, lactate, and VCD) was effectively maintained across different culture conditions (batch and fed‐batch) and may vary depending on the number of transfer learnings performed. Glutamine concentration monitoring could be improved by combining transfer learning with the development of a dedicated PLS model. This approach not only enhances the efficiency of implementing Raman spectroscopy for process monitoring but also broadens its applicability to diverse bioprocesses. Consequently, the study supports the notion that leveraging pre‐existing models can streamline the implementation of Raman spectroscopy tools in processes, offering substantial benefits in terms of both time and resource optimization in biomanufacturing.

Using this innovative method, it could be envisioned by equipment suppliers to directly integrate so‐called “embedded” models into Raman systems. With a transfer learning module, the tracking of parameters of interest could be configured by users through a library of pre‐existing models, which can be easily transferred to their process if the DS remains similar.

## AUTHOR CONTRIBUTIONS

**Schini Adèle**: Validation; writing — orginial draft; investigation, and methodology. **El Radi Hadi**: Conceptualization; data curation; visualization; writing — original draft; investigation; formal analysis, and methodology. **Cailletaud Johan**: Conceptualization; investigation; writing — original draft; methodology; visualization; formal analysis, and data curation. **Sanchez Célia**: Conceptualization; methodology; validation; writing — original draft; data curation; investigation, and formal analysis. **M. Gay Nathan**: Writing — original draft; methodology; validation; formal analysis, and data curation. **Ebel Bruno**: Resources; supervision; writing — review and editing, and project administration. **Guedon Emmanuel**: Project administration; supervision; resources; writing — review and editing. **Jourdainne Laurent**: Writing — review and editing; project administration; resources; supervision, and conceptualization.

## CONFLICT OF INTEREST STATEMENT

The authors declare no conflict of interest.

## Supporting information


**Figure S1:** Viability observed on the fed batch cultures from the input process.

**Figure S2:** Viability observed on the batch cultures from the target process.

**Figure S3:** Results of direct modeling on fed batch 3.

## Data Availability

The data that support the findings of this study are available from the corresponding author upon reasonable request.
