# Supplementary figures and images for: Innovative methodology for flexible monitoring of various bioprocesses by using pre‐existing Raman data coupled with automated transfer learning technique
Source: Biotechnol Prog. 2026 Feb 10;42(3):e70111. doi: 10.1002/btpr.70111 (PMC13266944; doi:10.1002/btpr.70111)

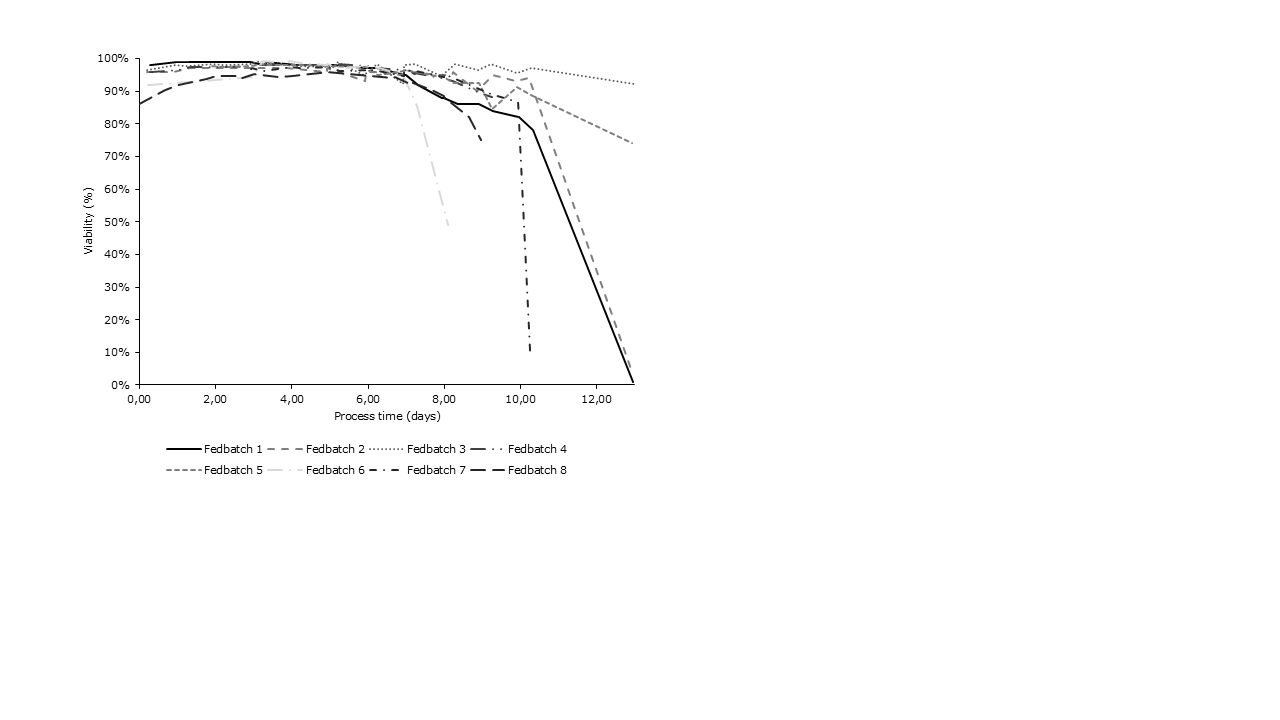

Supplement: Supplementary file 1 — Figure S1: Viability observed on the fed batch cultures from the input process. [file BTPR-42-e70111-s003.tif]

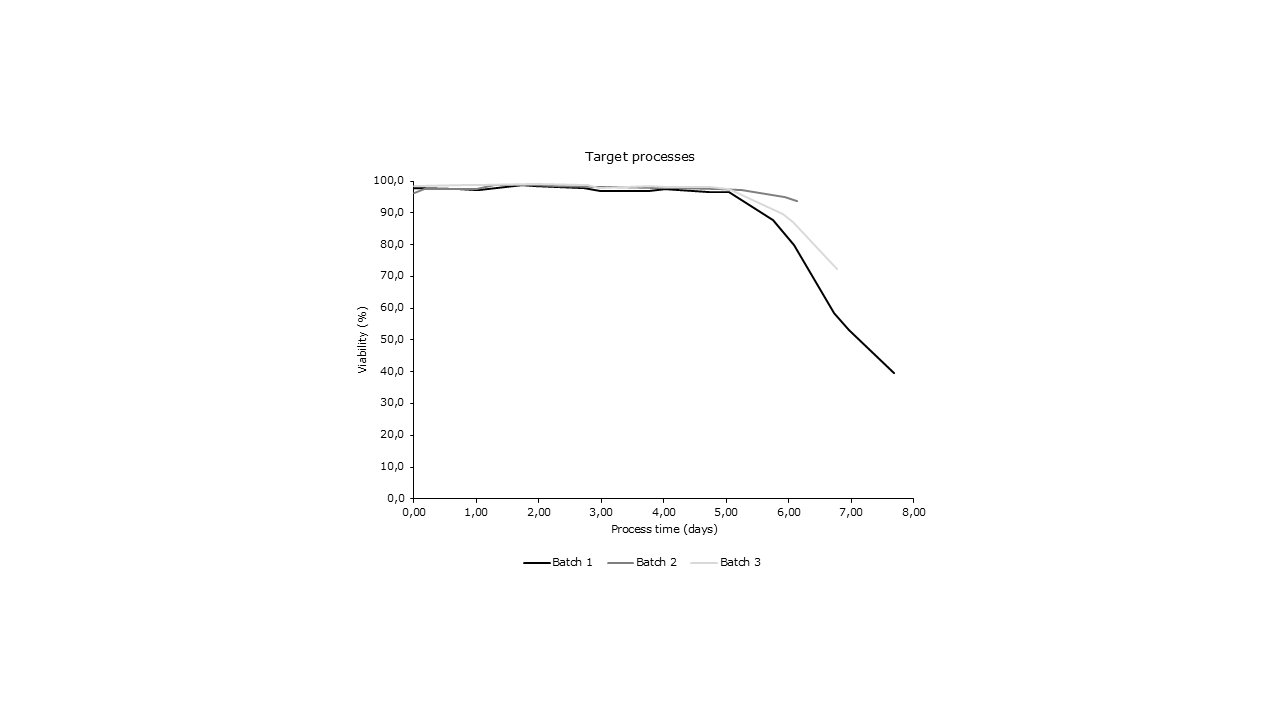

Supplement: Supplementary file 2 — Figure S2: Viability observed on the batch cultures from the target process. [file BTPR-42-e70111-s001.tif]

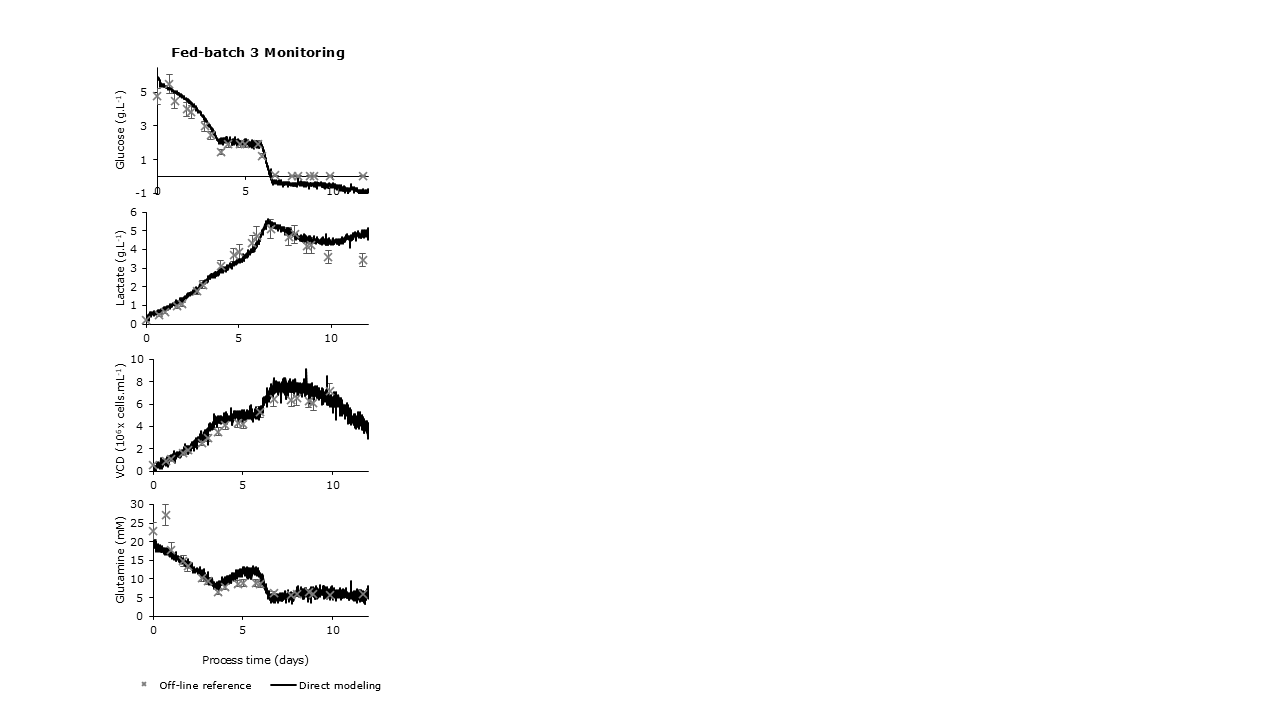

Supplement: Supplementary file 3 — Figure S3: Results of direct modeling on fed batch 3. [file BTPR-42-e70111-s002.tif]
